# Strain-Induced Quantum Spin Hall Effect in Two-Dimensional Methyl-Functionalized Silicene SiCH_3_

**DOI:** 10.3390/nano8090698

**Published:** 2018-09-07

**Authors:** Ceng-Ceng Ren, Wei-Xiao Ji, Shu-Feng Zhang, Chang-Wen Zhang, Ping Li, Pei-Ji Wang

**Affiliations:** School of Physics, University of Jinan, Jinan 250022, China; wziran@sina.com (C.-C.R.); sps_jiwx@ujn.edu.cn (W.-X.J.); sps_zhangsf@ujn.edu.cn (S.-F.Z.); ss_zhangcw@ujn.edu.cn (C.-W.Z.); ss_lip@ujn.edu.cn (P.L.)

**Keywords:** quantum spin hall effect, spin-orbital coupling, silicene, SiCH_3_

## Abstract

Quantum Spin Hall (QSH) has potential applications in low energy consuming spintronic devices and has become a researching hotspot recently. It benefits from insulators feature edge states, topologically protected from backscattering by time-reversal symmetry. The properties of methyl functionalized silicene (SiCH_3_) have been investigated using first-principles calculations, which show QSH effect under reasonable strain. The origin of the topological characteristic of SiCH_3_, is mainly associated with the s-p_xy_ orbitals band inversion at Γ point, whilst the band gap appears under the effect of spin-orbital coupling (SOC). The QSH phase of SiCH_3_ is confirmed by the topological invariant Z_2_ = 1, as well as helical edge states. The SiCH_3_ supported by hexagonal boron nitride (BN) film makes it possible to observe its non-trivial topological phase experimentally, due to the weak interlayer interaction. The results of this work provide a new potential candidate for two-dimensional honeycomb lattice spintronic devices in spintronics.

## 1. Introduction

Two-dimensional (2D) topological insulators (TIs) or Quantum Spin Hall (QSH) insulators are characterized by insulating bulk and metallic edge states [1,2,3,4,5,6]. Its gapless edge state is protected by time-reversal symmetry (TRS) [3,6]. Kane and Mele first presented the initial concept of the QSH insulator for grapheme [3,4], in which the effect of SOC opened a band gap at the Dirac point, but its bulk gap was too small (~10^−3^ meV) due to its rather weak SOC [3,7], resulting in the QSH effect observed only at extremely low temperature. In addition, various principles have been applied to silicone [8], germanene [9], stanene [10], plumbene [11], and III-V bilayer [12], and these 2D TIs have a similar honeycomb lattice to graphene. HgTe/CdTe and InAs/GaSb quantum wells (QWs) are a well-established system, their quantized conductance in the QSH effect have only been experimentally detected at the ultralow temperature, which greatly obstructs their potential application in quantum devices and spintronics [13,14,15,16].

Finding large bulk gap topological insulators for observing the spin transport at room temperature, as well as finding materials that are convenient to synthesize in experiments, is a trend in current topology development [17]. In addition, compatibility with current silicon-based electronic technology is also indispensable [18]. An effective way to achieve the QSH effect is starting from the atomic level and tuning the chemical bonding to induce the band inversion by SOC. New 2D TIs with a strong SOC, which are generally composed of relatively heavy elements, As [19], Bi [20], Sb [21], additionally, the group V films and group III-V materials GaAs and GaBi, have also been reported to be large-gap QSH insulators [22,23,24]. Recently, methyl-functionalized germanene (GeCH_3_) has been certified to be large-gap QSH insulators [18].

Furthermore, 2D SnCH_3_ films have been proposed to realize TIs with a band gap larger than 340 meV [25]. The orbital filtering effect (OFE) can be applied to design a QSH insulator, which is an effective way to enhance the bulk band gap of 2D materials [26,27,28]. Kaloni et al. proposed that the adsorption of small organic molecules can alter the electronic structure of silicone [29].

Silicon (Si) atom, the counterpart of group-IV carbon, is known for its unique electronic properties and prospects for future application. Here, based on first-principles calculations, we predicated a new QSH insulator in Si, by functionalized organic molecule group methyl. Our results revealed that the structural stability of SiCH_3_, can be confirmed by phonon spectrum. The external strains can tune the band gap of SiCH_3_ effectively, in which the s-p band inversion occurs and realizes a QSH insulator. In addition, we found that the SiCH_3_ on BN film makes it possible to support the non-trivial topological phase, due to the weak interlayer interaction. These results may provide a new candidate for designing large-gap QSH insulators, which is necessary for device applications in spintronics.

## 2. Computational Details and Methods

All density functional theory (DFT) [30] calculations were performed using the Vienna ab initio Simulation Package (VASP) [31]. Specifically, the Perdew-Burke-Ernzerhof (PBE) of generalized gradient approximation (GGA) was used to describe the exchange correlation energy [32,33], which was developed to calculate surface systems. The projector augmented wave (PAW) methods, processing the ion-electron interactions with the vdW interaction, were considered using the dispersion-correction functional of DFT-D3 [34,35]. The energy cutoff of the plane wave basis set was 500 eV, and the convergence criterion was 10^−6^ eV between our self-consistent calculation steps. A 7 × 7 × 1 Monkhorst-Pack uniform k-grid was used in the 2D Brillouin zone, for geometry optimizations and electronic calculations, as described in Reference [36]. The SOC was included in calculations. The vacuum region was set to 20 Å, to eliminate any artificial interaction between neighboring slabs, as explained in References [37,38]. The phonon spectra were calculated along high symmetry lines, using a density functional perturbation theory method, implemented in PHONOpy code [39]. In addition, all atomic positions and the size of the unit cell were optimized using the conjugate gradient method until the atomic forces were less than −0.01 eV Å^−1^. To further evaluate thermal stability, we used a 5 × 5 × 1 supercell to perform the ab initio molecular dynamics (AIMD) simulations.

## 3. Results and Discussions

### 3.1. Electronic Structure

**Figure 1:**
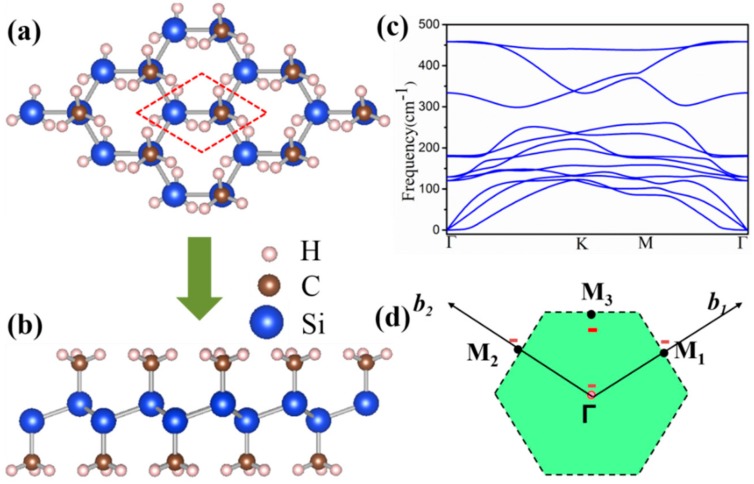
(**a**) Top and (**b**) side views of the schematic structures of silicene (SiCH_3_) monolayer; (**c**) Phonon spectrum and (**d**) the area of Brilioun zone of SiCH_3_ monolayer.

Figure 1a,b proposes a schematic diagram of the structure of a methyl double-sided functionalized silicone, each cell containing two Si atoms and two methyl groups. The decoration of the chemical functional groups provides more possibilities, and the methyl modified silicone makes a strong bond between the Si atom and CH_3_. As for SiCH_3_, a large buckle height (h = 0.78 Å) is the main feature distinct from the planer grapheme [40,41]. The buckle enhances the p-p coupling between p-orbitals of Si atoms, in coexistence with certain overlap between p and σ orbitals. The buckle height plays an extremely important role in the engineering of electronic properties. In addition, the structure belongs to the P3 layer group with optimized lattice constants a = b = 3.90 Å, the Si–Si and Si–C being 2.39 and 1.92 Å, respectively.

**Figure 2:**
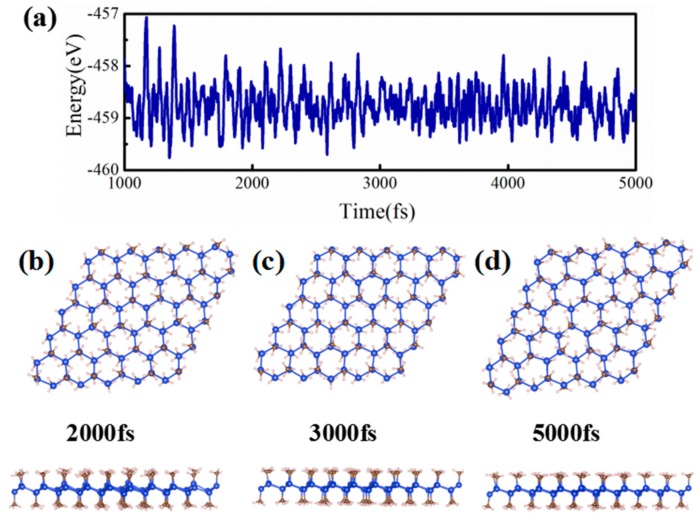
(**a**) Variations of the free energy from 2000 to 5000 fs, during ab initio molecular dynamics simulations (AIMD) at the temperature of 300 K for SiCH_3_; (**b**–**d**) indicated the snapshot of molecular dynamics (MD)simulation of the structure in 2000, 3000, and 5000 fs, respectively.

In order to prove the stability of SiCH_3_ monolayer, its formation energy has been computed defined by:

(1) $$\Delta E=E\left({\mathrm{SiCH}}_{3}\right)-E(\mathrm{Silicene})-{E(\mathrm{CH}}_{3})$$

where E(SiCH_3_) and E(Silicene) are the total energies of functionalized silicene and pristine silicene, respectively, whilst E(CH_3_) is the chemical energy of methyl. The formation energy ΔE was calculated and it was −5.35 eV/atom for SiCH_3_, indicating there was no phase separation in this system. To further confirm the structural stability, the phonon spectrum along the highly symmetric directions was calculated, as shown in Figure 1c. There was no mode with imaginary frequencies in the Brillouin zone, which indicated that SiCH_3_ was stable. Furthermore, the thermal stability of SiCH_3_ was evaluated by performing the ab initio molecular dynamics (AIMD) simulations. Here, the SiCH_3_ monolayer was subjected to the molecular dynamics (MD) simulations, at a setting temperature of 300 K. We plotted the snapshots of SiCH_3_ monolayer at 2000, 3000, and 5000 fs for these simulations, as shown in Figure 2. In short, when the temperature reached 300 K, the honeycomb skeleton of SiCH_3_ monolayer had almost no distortion and no bond breaking arose. The above results revealed that the SiCH_3_ monolayer has very good thermal stability and maintains its structural integrity at room-temperature environment, providing a possibility for experimental preparation and application.

**Figure 3:**
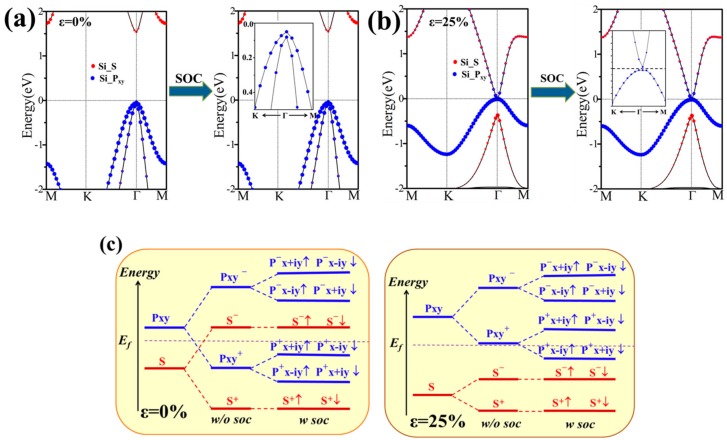
The orbital–resolved band structures of SiCH_3_ monolayer structures without and with spin-orbital coupling (SOC) (**a**) 0% and (**b**) 25% strain; (**c**) Schematic diagram of the evolution from the atomic s and p_xy_ orbitals of Si at Γ point. The Fermi level is indicated by horizontal dashed lines.

The band structures without SOC and with SOC were calculated for SiCH_3_ monolayer and shown in Figure 3, where the red and blue color represents the s and p_xy_ orbital. Different from the Silicene without methyl modification, the decorated chemical functional group strongly hybridized with the dangling bonds of the p_z_ orbital in silicene, which led the p_z_ orbital to move away from the Fermi level. It was more conducive to our regulation of the electronic properties of the material. In the absence of SOC (Figure 3a), the band structure exhibits a semiconductor character with a direct band gap of 1.59 eV at Γ point. By projecting the energy bands onto different atomic orbitals, we found that the energy band was mainly composed of the s and p_xy_ orbitals of Si atoms near the Fermi level. The valence band maximum (VBM) at Γ point was mainly contributed by the p_xy_ orbitals of Si atoms with the characteristic of the bonding state, whereas the conduction band minimum (CBM) was mainly contributed by the s orbital of Si atoms and was the anti-bonding state. When SOC was included, the energy degeneracy of p_xy_ orbits at Γ point lifted significantly, and the degenerate level was split by a gap of 11 meV (Figure 3a). The energy degeneracy at the Γ point could be clearly seen through the enlarged view. There was no inverted band order in SiCH_3_ (*ε* = 0%), suggesting that it was a trivial insulator.

### 3.2. Strain Properties

**Figure 4:**
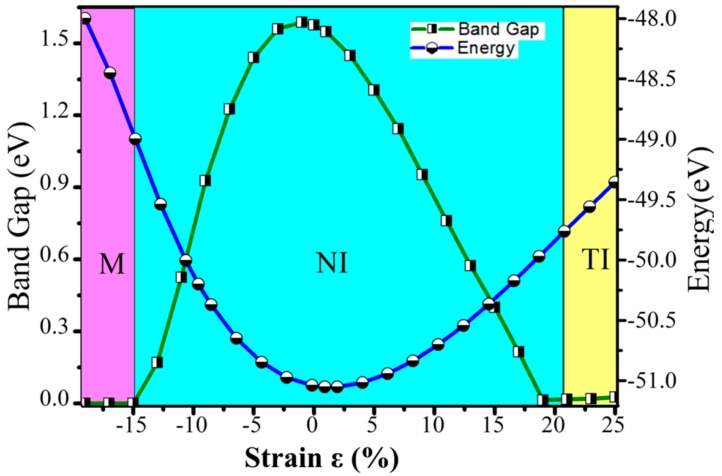
The energy and the band gap calculated for a SiCH_3_ monolayer, as a function of external strain.

After obtaining the structure of SiCH_3_, mechanical strain changes the chemical bond strength and thus affects the energy band inversion. Here, various biaxial strains were applied to the SiCH_3_ lattices, and we defined the biaxial strain *ε* = (*a* − a_0_)/a_0_, where *a* and a_0_ were the tensile and equilibrium strained lattice constant. Additionally, a negative *ε* presents the compressive strain, whilst a positive value indicates the tensile stress. Figure 4 gives a function of strain *ε* from −19–25% about the band gap and energy of SiCH_3_. In this range, SiCH_3_ can be summarized with some few interesting features. One can see clearly that the energy of the SiCH_3_ presents continuous variation with the change of the strain, confirming that the applied strain was elastic, which could be attributed to the field of elastic strain engineering (ESE) [42,43]. In addition, the band gap decreases monotonically with the lattice constant increasing, which is inconsistent with the case of graphene monolayer [44]. When the strain is large enough, the band gap is closed; continue to increase the strength of the stress, and a small band gap occurs, suggesting a transition from trivial semiconductor to topological phase, and it shows that the strain can modulate the topological properties.

### 3.3. Topological Properties

To conduct a preliminary analysis of the topological properties of the material, we took the corresponding band structures of SiCH_3_ (*ε* = 25%) monolayer as an example. This is schematically illustrated in two stages in Figure 3b. Without SOC, the band structure turns to the gapless states with the Fermi level crossing two degenerated p_xy_ orbitals, and compared with p_xy_ orbitals, the s orbital has a lower energy. When the SOC is included, the degeneracy of p_xy_ orbitals are lifted with opening of a gap of 26 meV. The band gap change is relatively easy to observe from the enlarged view. An inverted order of s orbital and p_xy_ orbitals appeared, which indicated the possibility of a topological phase transition. This inversion mechanism is the similar to some of the topological materials that have been reported, such as the HgTe quantum well. In short, the s-p type band inversion can be experienced under the mechanical strain, and hence lead to a non-trivial TI state.

To demonstrate the topological properties of SiCH_3_ monolayer, we calculated the Z_2_ invariants *ν*, based on the scheme proposed by Fu-Kane’s [45], the equation can be expressed by

(2) $$\delta (K_{i})=\prod_{m=1}^{N}\xi_{2m}^{i},{\left(-1\right)}^{\nu }=\prod_{i=1}^{4}\delta \left(K_{i}\right)=\delta \left(\Gamma \right)\delta {\left(M\right)}^{3}$$

where *δ* is the product of parity eigenvalues at four time reversal invariant momentum (TRIM), *ν* is the number of the occupied bands, and *ξ* = ±1 are the parity eigenvalues. According to the Z_2_ classification, *ν* = 1 represents a topologically non-trivial phase and *ν* = 0 shows trivial band topology. We calculated the Z_2_ topological invariant *ν* at four TRIM points *K_i_*, involved one *Γ* point, and three *M* points in the Brillouin zone (BZ), as shown in Table 1. When ε = 25%, the *δ* at these three symmetry points: M_1_ (0.5, 0.0), M_2_ (0.0, 0.5), and M_3_ (0.5, 0.5) are both −1, whilst at the Γ (0.0, 0.0) they show +1, yielding a Z_2_ of SiCH_3_ (ε = 25%) monolayer of 1 [46]. This means that it is a topological insulator.

**Figure 5:**
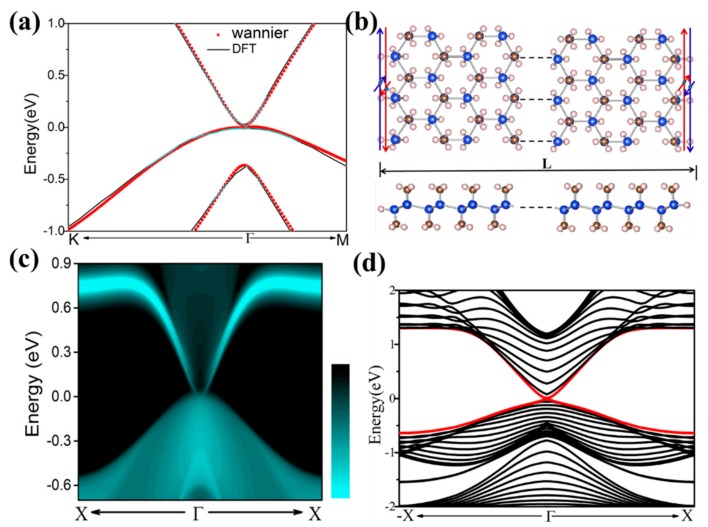
Electronic structure and its corresponding edge state of SiCH_3_ with 25% tensile biaxial strain. (**a**) Comparison of band structures for SiC density functional theory (DFT) H_3_, calculated by DFT (red lines) and Wannier functions method (blue dots); (**c**) Show the Dirac edge states. The Fermi level is set to zero; (**b**,**d**) the model and spectrum of a finite slab of SiCH_3_. The red and black horizontal arrows represent the spin-up and spin-down polarized currents in the opposite direction.

A notable feature of the QSH insulator is the presence of a one-dimensional helical in the bulk band gap, with the spin momentum locked. The opening gap and band inversion caused by the SOC near the fermi level, means that there is a possibility of the QSH effect, which has gapless edge states protected by TRS. To see the helical edge states explicitly, their edge states of SiCH_3_ were calculated using the Wannier90 package, with the maximally Localized Wannier functions (MLWFs), as described in Reference [47]. Figure 5a shows the band structures calculated by DFT and MLWF with 25% tensile strain, respectively. They were well matched to each other, which indicated the reliability of the MLWF calculation. The total edge density of states (DOS) were plotted in Figure 5c. We could clearly see two one-dimensional gapless edge states, connecting the conduction band and valence band of SiCH_3_ (*ε* = 25%). To analyze the origin of the dangling bond state, we took SiCH_3_ with the external strain *ε* = 25% by introducing a zigzag-shaped and sufficiently wide nanoribbon to introduce edges on the silicon; the interaction between the edge states of both sides is avoided, as shown in Figure 5b. The dangling bonds were eliminated by passivating all edges of the Si atoms with hydrogen atoms. The calculated band structure of the nanoribbon is displayed in Figure 5d. We could clearly observe that the same edges led to two obviously degenerate Dirac cones, located at the opposite edges. The gapless edge states (red lines) appear in the gap and cross linearly at the Γ point, the Dirac point at the Γ point is located in the band gap with a high velocity of ~1.0 × 10^5^ m/s, and the HgTe/CdTe [13,14] quantum well possesses a higher velocity of 5.5 × 10^5^ m/s, than that of the InAs/GeSb [15,16] quantum well (3.0 × 10^4^ m/s). These results indicated that SiCH_3_ is an ideal 2D TI and provide new candidate materials for novel quantum electronic devices with low energy dissipation.

To get a clear understanding of the origin of topological non-triviality, we presented the methodical band evolution at Γ point for SiCH_3_ in Figure 3c. Near the Fermi level, the Si-s and Si-p_xy_ states dominate the relevant bands. Under the effect of crystal field splitting, the chemical bonding between Si-Si atoms forms bonding states and anti-bonding states for the s and p_xy_ orbitals, which we denote as |s^±^> and |p_xy_^±^>, with the superscript + and − representing the parities of corresponding states, respectively. As displayed in Figure 3c, the bands are near the Fermi level due to the |s^−^> and |p_xy_^+^>, and the |p_xy_^+^> is below |s^−^> under the effect of crystal field. At a strain of *ε* = 25%, the |p_xy_^+^> level shifts above the |s^−^>, which leads to the s-p type band inversion. When the SOC is turned on, the degeneracy of the level splits into |p, ±3/2> states with a total angular momentum j = 3/2 and |p, ±1/2> with a total angular momentum j = 1/2, creating an energy gap at the Γ point. A remarkable feature of the band component is that the SOC is only to open a band gap but does not induce the band inversion. The conclusion above, is that the SiCH_3_ monolayer with *ε* = 25% is a QSH insulator.

### 3.4. Si/BN Heterostructures

**Figure 6:**
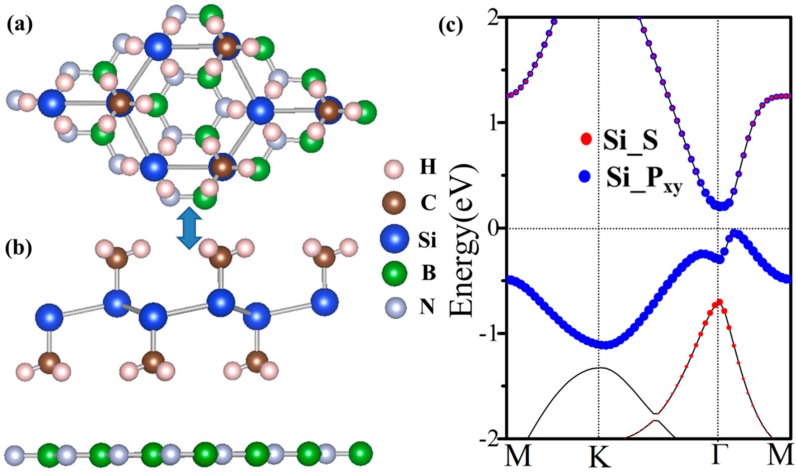
(**a**) Top and (**b**) side views of the epitaxial growth SiCH_3_ on 2 × 2 BN substrate; (**c**) The corresponding energy band structure with SOC.

From an experimental point of view, the growth of 2D materials is the ultimate goal. The mechanical exfoliation and molecular beam epitaxy (MBE) are relatively mature. The synthesis of silicone were reported using MBE. The substrate materials are indispensable for experimental preparation. Hexagonal boron nitride is a 2D large gap insulator with high dielectric constant, we chose BN-2 × 2 (5.06 Å) as the substrate to support the SiCH_3_ single layer [48,49]. In this work, it was exhibited as an acceptable lattice mismatch (~3.6%), showing that it was possible for SiCH_3_ to grow on the h-BN substrate. We used the DFT method (optB88-vdw) to describe 2D HTS (heterostructure), to avoid erroneous estimation of van der Waals (vdW) interactions [50]. The expected band structure with SOC is shown in Figure 6. In these weakly coupled QW structures, the SiCH_3_ monolayer remained semiconducting, whilst the distance between adjacent layers was 3.52 Å. The binding energy was obtained to be −10.54 meV per unit cell, indicating a weak interaction between SiCH_3_ and BN sheet, and demonstrating a typical 2D vdW HTS. Bader charge analysis indicated that the Fermi levels were dominantly attributed to the SiCH_3_ monolayer. The differences were little between them, compared to SiCH_3_ without substrate. It is obvious that SiCH_3_ is a robust QSH insulator and the topological properties are not affected by the substrate. This work may provide new candidates for the design and manufacture of QSH insulators, based on 2D cellular lattices in spintronics.

## 4. Conclusions

In this work, we predicted SiCH_3_ monolayer to a new 2D QSH insulator, based on first-principle calculations. It was found that the external strain can induce a topological phase transition in SiCH_3_ monolayer. The origin of topological characteristics of SiCH_3_ is mainly associated with the s-p_xy_ orbitals band inversion of Si atoms, and it opened a band gap with the effect of SOC, rather than change the band order. Moreover, the Z_2_ invariant and topologically protected edge states confirmed the topological characteristic of the SiCH_3_ monolayer. The SiCH_3_ supported by BN film makes it possible to observe its non-trivial topological phase experimentally, due to the weak interlayer interaction. The results of this work provide new potential candidates for two-dimensional honeycomb lattice spintronic devices in spintronics.

## Figures and Tables

**Table 1:** Parities of occupied spin-degenerate bands at the time reversal invariant momentum (TRIM) Points for silicene (SiCH_3_). Here, we show the parities of 11 occupied spin-degenerate bands for SiCH_3_ (*ε* = 0% and *ε* = 25%). Positive and negative signs denote even and odd parities, respectively.

*Γ_i_*	Parity of ζ_2*n*_ of Occupied Bands	*δ_i_*	*Γ_i_*	Parity of ζ_2*n*_ of Occupied Bands	*δ_i_*
(0.0, 0.0)	+ − + − + + − − − + +	−	(0.0, 0.0)	+ − + − + + + − − + +	+
(0.5, 0.0)	+ − + − + − + − − + +	−	(0.5, 0.0)	+ − + − + − + − − + +	−
(0.0, 0.5)	+ − + − + − + − − + +	−	(0.0, 0.5)	+ − + − + − + − − + +	−
(0.5, 0.5)	+ − + − + − + − − + +	−	(0.5, 0.5)	+ − + − + − + − − + +	−
*ε* = 0%	Z_2_ topological invariant	*ν* = 0	*ε* = 25%	Z_2_ topological invariant	*ν* = 1
